# Tetrameric hotdog‐fold structure and catalytic mechanism of the *Sa* PaaI thioesterase from *Staphylococcus aureus*

**DOI:** 10.1002/pro.70583

**Published:** 2026-04-27

**Authors:** Yogesh Khandokar, Parul Srivastava, Renate H. M. Schwab, Ashish Sethi, Naveen Vankadari, Jade K. Forwood

**Affiliations:** ^1^ Australian Synchrotron, Australian Nuclear Science & Technology Organisation Clayton Victoria Australia; ^2^ School of Biomedical Sciences Charles Sturt University Wagga Wagga New South Wales Australia; ^3^ Gulbali Institute Charles Sturt University Wagga Wagga New South Wales Australia; ^4^ Department of Biochemistry and Pharmacology The University of Melbourne Parkville Victoria Australia; ^5^ Department of Biochemistry and Molecular Biology Monash University, Wellington Road Clayton Victoria Australia; ^6^ School of Agricultural, Environmental and Veterinary Sciences Charles Sturt University Wagga Wagga New South Wales Australia

**Keywords:** acyl‐CoA thioesterase, benzoyl‐CoA, catalytic dyad, conformational gating, hotdog fold, *Sa*PaaI, *Staphylococcus aureus*, TE11 family

## Abstract

Acyl‐CoA thioesterases hydrolyse thioester bonds to release free fatty acyl chains and coenzyme A (CoA), thereby regulating lipid metabolism, signaling, and membrane homeostasis. Here, we present the structural and functional characterization of the Paal‐like thioesterase SAV0944 (*Sa*PaaI) from *Staphylococcus aureus*. *Sa*PaaI adopts a class‐II hotdog fold comprising six β‐strands wrapped around a central α‐helix and assembles as a tetrameric dimer‐of‐dimers, as determined by x‐ray crystallography and analytical size‐exclusion chromatography. Enzyme assays using a panel of acyl‐CoA substrates identify benzoyl‐CoA as the preferred substrate. Guided by structural alignment with homologous thioesterases, Gln32 and Glu47 were identified as essential catalytic residues; alanine substitutions at either position abolished activity without perturbing the global fold. The crystal structure revealed asymmetric CoA binding, with only two monomers in the tetramer displaying well‐defined ligand density. Comparison of CoA‐bound and ligand‐free monomers showed that Gln32 undergoes a ~102° conformational rotation that opens the substrate tunnel in the bound state, whereas the corresponding unliganded monomers adopt a closed conformation that sterically occludes the pocket. This mutually exclusive positioning of Gln32 within each dimer provides structural evidence for half‐of‐the‐sites behavior, suggesting that *Sa*PaaI employs a ligand‐induced gating mechanism that modulates substrate access. Together, these findings establish *Sa*PaaI as a benzoyl‐CoA–selective thioesterase with a noncanonical catalytic configuration and uncover an asymmetric, Gln32‐dependent gating mechanism that contributes to substrate specificity in this essential *S. aureus* enzyme.

## 1 INTRODUCTION

Thioesterases (TEs) are a diverse group of enzymes that hydrolyse thioester bonds in lipid and metabolic pathways, generating free fatty acids and coenzyme A (CoA). They play central roles in fatty‐acid biosynthesis, β‐oxidation, and regulation of metabolic intermediates, as well as in detoxification pathways and the biosynthesis of lipid‐derived secondary metabolites (Black et al., 2000; Cantu et al., 2010; Hunt & Alexson, 2002; Hunt & Alexson, 2008; Kirkby et al., 2010; Leesong et al., 1996). Based on their structural folds and catalytic mechanisms, TEs are classified broadly into two major families: the α/β‐hydrolase family and the hotdog‐fold family (Angelini et al., 2008; Forwood et al., 2007). Hotdog‐fold thioesterases are widespread across bacteria, archaea, and eukaryotes, and participate in crucial processes including lipid homeostasis, quorum‐sensing metabolite turnover, and stress‐response regulation (Badger et al., 2005; Benning et al., 1998; Kunishima et al., 2005; Thoden et al., 2003).

The hotdog fold comprises a characteristic arrangement of five to seven β‐strands wrapped around a long central α‐helix, forming either homodimeric or higher‐order oligomeric assemblies (Angelini et al., 2008; Khandokar et al., 2016). Many bacterial hotdog‐fold thioesterases function as dimers of dimers, generating a composite active site at the dimer interface. Their substrate specificity varies widely and includes short‐chain, branched, aromatic, and long‐chain acyl‐CoAs. Structural studies have shown that subtle changes in active‐site residues or tunnel geometry can significantly influence chain‐length preference, catalytic rate, and physiological function (Angelini et al., 2008; Badger et al., 2005; Marfori et al., 2011).

*Staphylococcus aureus* is an opportunistic Gram‐positive pathogen responsible for a wide range of difficult‐to‐treat infections, including bacteraemia, pneumonia, endocarditis and device‐associated biofilm formation (Fowler & Proctor, 2014; Lowy, 1998). Its capacity to adapt to diverse host environments relies in part on metabolic flexibility, including modulation of fatty‐acid utilization and acyl‐CoA turnover. Although several *S. aureus* metabolic enzymes have been characterized structurally, many predicted thioesterases remain poorly defined and their substrates and cellular roles are still unknown.

SAV0944, hereafter referred to as *Sa*PaaI, is a putative bacterial acyl‐CoA thioesterase belonging to the Paal‐like clade of hotdog‐fold enzymes. Homologues in other bacteria have been shown to hydrolyse aromatic or medium‐chain acyl‐CoA substrates, suggesting potential roles in detoxification of metabolic by‐products, catabolism of environmental aromatic compounds, or regulation of lipid‐derived signaling pathways (Badger et al., 2005; Koshland, 1958; Swarbrick et al., 2015). Despite this, *Sa*PaaI has not previously been structurally or functionally characterized.

Here, we present the structural and biochemical characterization of *Sa*PaaI protein from *S. aureus* subsp. *aureus* Mu50. We solved its crystal structure, analyzed its oligomeric assembly, determined substrate preference using a panel of acyl‐CoA molecules, and identified key catalytic residues through mutagenesis. Our data show that *Sa*PaaI adopts a canonical class‐II hotdog fold yet exhibits unique active‐site features that explain its preference for benzoyl‐CoA. Furthermore, we demonstrate that Gln32 and Glu47 are essential for catalysis and contribute to a gating mechanism that modulates substrate access to the active site. Unexpectedly, the structure also reveals asymmetric ligand occupancy within the tetramer, suggesting that *Sa*PaaI may utilize a half‐of‐the‐sites regulatory mechanism. Together, these findings provide the first detailed structural and mechanistic insights into *Sa*PaaI and establish a foundation for understanding aromatic‐acyl‐CoA metabolism in *S. aureus*.

## 2 RESULTS AND DISCUSSION

### 2.1 Overall structure

A *Sa*PaaI protein crystal obtained in presence of CoA diffracted to 2.0 Å resolution in space group P1211 and was used for structure determination (Figure S3; Table 1). Diffraction images were integrated using iMosflm (Battye et al., 2011), and the structure was solved by molecular replacement using chain A of *E. coli* YdiI (PDB ID: 1SBK; unpublished; 38% sequence identity), with the N‐terminal 23 residues (disordered region) removed from the search model. A Matthews coefficient of 2.18 Å^3^/Da supported the presence of four *Sa*PaaI molecules in the asymmetric unit (Matthews, 1968; Winn et al., 2011), and all four monomers were built and refined in Coot (Emsley & Cowtan, 2004). The four monomers in the asymmetric unit adopt highly similar conformations, with a maximum Cα RMSD of 0.55 Å, indicating minimal structural variation. The final refined model displayed excellent stereochemistry, with R_work = 17.3%, R_free = 22.0%, and no Ramachandran outliers (Table 1). Coordinates and structure factors have been deposited in the Protein Data Bank under accession code 4M20.

**TABLE 1:** Data collection and refinement statistics of *Sa*PaaI*‐*WT, *Sa*PaaI‐2Gln^32^Ala, and *Sa*PaaI‐2Glu^47^Ala.

	*Sa*PaaI‐WT	*Sa*PaaI‐Gln^32^Ala	*Sa*PaaI‐Glu^47^Ala
Wavelength (Å)	0.9537	0.9537	0.9537
Resolution range (Å)	30.14–2.0 (2.07–2.0)	29.75–2.0 (2.07–2.0)	46–2.9 (3.0–2.9)
Space group	P 1 21 1	P 1 21 1	P 1 21 1
Unit cell	44.05 89.05 60.74 90100.53 90	44.64 90.85 65.15 90 99.02 90	44.22 91.57 65.74 90 99.52 90
Unique reflections	30,911 (3090)	34,169 (3386)	10,339 (1016)
Multiplicity	3.5 (3.5)	7.4 (7.3)	7.3 (6.8)
Completeness (%)	99.20 (99.39)	98.41 (97.64)	89.69 (89.99)
Mean I/sigma(I)	10.80 (3.78)	18.25 (6.22)	9.20 (1.82)
Wilson B‐factor	32.37	26.75	44.69
R‐merge	0.033 (0.173)	0.019 (0.108)	0.074 (0.391)
R‐work	0.173 (0.237)	0.191 (0.248)	0.217 (0.313)
R‐free	0.220 (0.293)	0.234 (0.317)	0.262 (0.366)
Refined model			
Number of non‐hydrogen atoms	3983	3905	3656
Macromolecules	3695	3682	3656
Ligands	96		
Water	192	223	0
Protein residues	483	484	481
RMS deviation			
RMS (bonds)	0.016	0.014	0.01
RMS (angles)	1.4	1.5	1.47
Ramachandran favored	99	97	98
Ramachandran allowed	1	3	2
Ramachandran outliers	0	0	0
Average B factor (Å^2^)	42.3	32.6	42.3
Macromolecules	41.2	32.3	42.3
Ligands	81.7		
Solvent	44	36.4	
PDB	4M20	4YBV	5EP5

*Note*: Statistics for the highest‐resolution shell are shown in parentheses.

The asymmetric distribution of bound and unbound monomers within the tetramer suggests that *Sa*PaaI may exhibit half‐of‐sites behavior, where only a subset of subunits is competent for ligand binding at a given time. Such partial occupancy has been observed in several symmetric oligomeric enzymes and is often associated with regulatory or cooperative mechanisms (Monod et al., 1965; Koshland et al., 1966). In the case of *Sa*PaaI, CoA binding to only two monomers may reflect an intrinsic asymmetry that stabilizes specific conformational states required for crystallization.

### 2.2 Topology and overall fold

**FIGURE 1:**
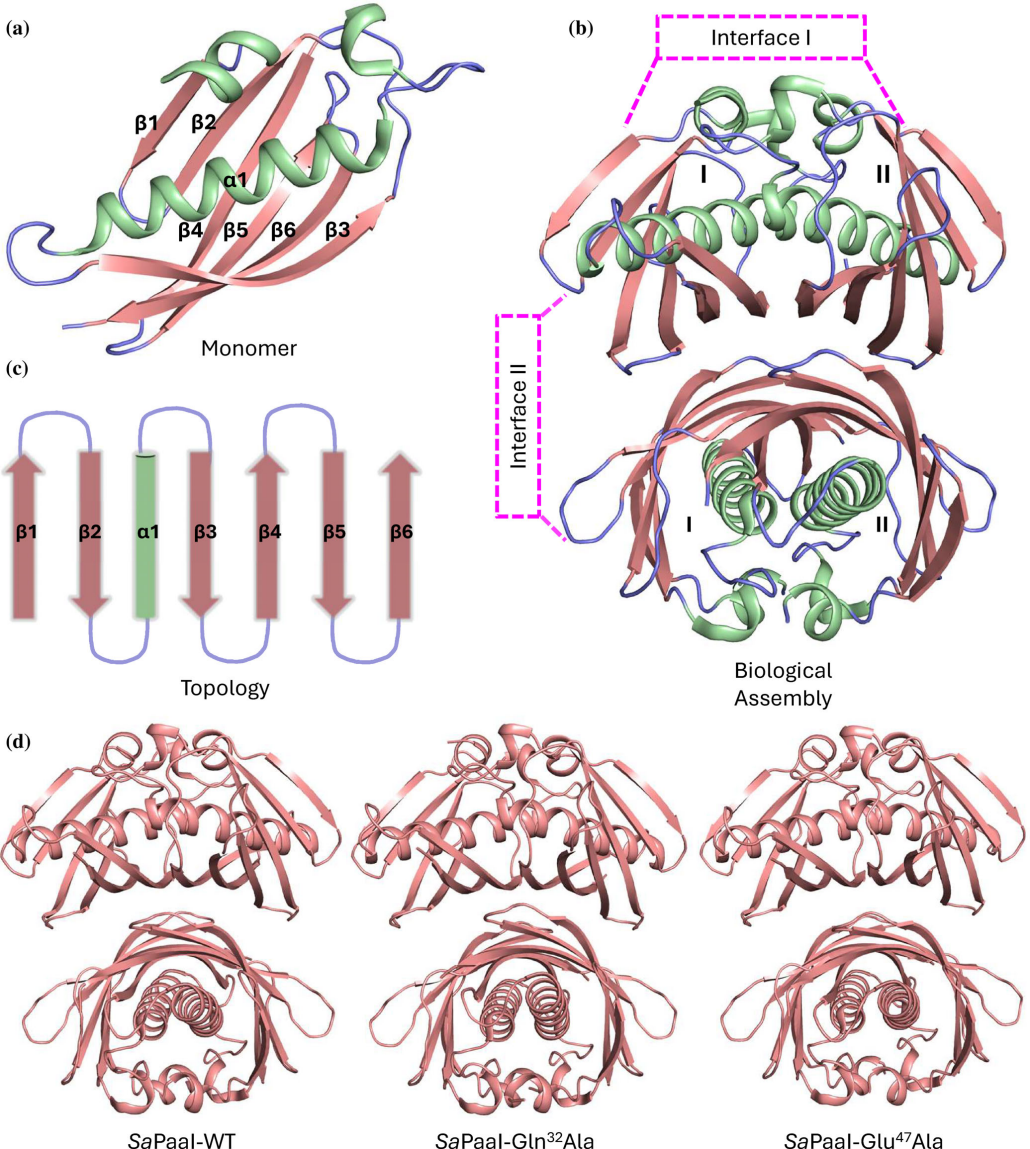
Structure and topology of SaPaaI and variants. (a) Cartoon representation of the SaPaaI monomer showing six β‐strands (Salmon) wrapped around a central α‐helix (PaleGreen), forming the characteristic class‐II hotdog fold. (b)Side and Top view showing the tetrameric assembly of SaPaaI, shown as a dimer of double‐hotdog domains representing the biologicalunit (2 monomers are labelled as IandII). (c)Topology diagram of SaPaaI, illustrating the arrangement of secondary‐structure elements (β1–β2–α1 β3–β4–β5–β6). (d) Overlay of tetrameric assemblies of wild‐type SaPaaI and the Gln32Ala and Glu47Ala variants. The hotdog‐fold architecture and quaternary structure are preserved in all constructs, confirming that loss of activity arises from catalytic‐site disruption rather than structural alteration.

*Sa*PaaI adopts a canonical class‐II hotdog fold with the secondary structure arrangement β1–β2–α1–β3–β4–β5–β6 (Figure 1a–c). The protein consists of six β‐strands wrapped around a single central α‐helix comprising 21 amino acids. The β‐strands range from 6 to 12 residues in length, forming the characteristic curved β‐sheet that embraces the central helix. This arrangement generates a typical hotdog‐like architecture. Analysis using the PISA server (Proteins, Interfaces, Structures and Assemblies) (Krissinel & Henrick, 2007) and analytical SEC both indicate that the tetrameric assembly represents the biological unit of *Sa*PaaI (Figure S1B). The tetramer is arranged as a dimer of double‐hotdog domains, typical of many bacterial acyl‐CoA thioesterases (Figure 1b). Each monomer protein exhibits a class‐II hotdog topology (β–β–α–β–β–β–β) with RMSD values below 1.5 Å relative to *Sa*PaaI (Figure 1c).

Similar to the wild‐type enzyme, variants crystallized in several conditions, and where possible, crystals were harvested in different crystallization conditions to confirm that the mutations do not alter the structure, but also that different salt, pH, and/or crystallization packing do not influence the structure. The structures of variants of *Sa*PaaI, Gln32Ala, and Glu47Ala were solved in the P1211 space group; coordinates and structural factors were deposited to PDB and obtained PDB codes 4YBV and 5EP5 respectively. The analysis of tertiary and quaternary structures of variants Gln32Ala and Glu47Ala revealed the similar hotdog architecture. Detailed statistical values of refinement and modeling are listed in Table 1. The analysis of the variant's structures revealed similar hotdog fold conformations to that observed in wild‐type *Sa*PaaI structure, with the greatest RMSD of 0.61 Å (*Sa*PaaI wild type with Gln32Ala) and 0.46 Å (*Sa*PaaI wild type with Glu47Ala) (Figure 1d).

### 2.3 Oligomeric interfaces

The *Sa*PaaI tetramer buries a total surface area of 10,458 Å^2^ within a combined interface area of 18,070 Å^2^, forming two distinct interaction surfaces. The Interface I (A:B and C:D) exists within each double‐hotdog dimer. This interface contributes most to tetramer stability, supported by 23 hydrogen bonds and four salt bridges (Table 2). Interface II (A:D and B:C) connects the two double‐hotdog dimers. This interface is smaller, containing six hydrogen bonds, and provides additional but lesser stabilization of the quaternary structure. These extensive networks of hydrogen bonds and salt bridges collectively stabilize the tetrameric form of *Sa*PaaI (Figure 1b; Table 2).

**TABLE 2:** PISA analysis showing interaction within interface‐I and interface‐II of SaPaaI.

Interface‐I			
H‐bonds	Chain	Dist (Å)	Chain
1	A:THR 7[OG1]	2.84	B:LYS 29[O]
2	A:LEU 4[N]	3.17	B:LYS 31[O]
3	A:HIS 3[N]	3.46	B:LYS 31[O]
4	A:HIS 74[NE2]	3.23	B:GLU 47[OE1]
5	A:GLY 40[N]	3.41	B:GLU 47[OE1]
6	A:HIS 38[NE2]	2.87	B:THR 48[OG1]
7	A:GLN 32[NE2]	3.56	B:SER 51[OG]
8	A:ASN 73[ND2]	3.83	B:LEU 68[O]
9	A:HIS 74[N]	2.92	B:LEU 68[O]
10	A:ALA 72[N]	3.86	B:GLU 69[OE2]
11	A:ALA 72[N]	2.82	B:MET 70[O]
12	A:MET 70[N]	2.87	B:ALA 72[O]
13	A:LYS 31[O]	3.62	B:HIS 3[N]
14	A:LYS 31[O]	3.06	B:LEU 4[N]
15	A:LYS 29[O]	2.75	B:THR 7[OG1]
16	A:THR 48[OG1]	2.91	B:HIS 38[NE2]
17	A:GLU 47[OE1]	3.49	B:GLY 40[N]
18	A:ALA 72[O]	2.89	B:MET 70[N]
19	A:MET 70[O]	2.83	B:ALA 72[N]
20	A:GLU 69[OE2]	3.78	B:ALA 72[N]
21	A:LEU 68[O]	3.83	B:ASN 73[ND2]
22	A:LEU 68[O]	2.91	B:HIS 74[N]
23	A:GLU 47[OE1]	3.05	B:HIS 74[NE2]

Salt bridges	Chain	Dist (Å)	Chain
1	A:HIS 74[NE2]	3.23	B:GLU 47[OE1]
2	A:HIS 74[NE2]	3.22	B:GLU 47[OE2]
3	A:GLU 47[OE1]	3.05	B:HIS 74[NE2]
4	A:GLU 47[OE2]	3.27	B:HIS 74[NE2]

Interface‐II			
H‐bonds	Chain	Dist(Å)	Chain
1	A:ARG 115[NH1]	2.73	D:ASN 71[OD1]
2	A:ARG 115[NE]	2.58	D:ASN 73[OD1]
3	A:ASN 73[ND2]	2.71	D:THR 117[OG1]
4	A:ASN 71[OD1]	2.78	D:ARG 115[NH1]
5	A:ASN 73[OD1]	2.71	D:ARG 115[NE]
6	A:THR 117[OG1]	2.81	D:ASN 73[ND2]

The substantial dimer–dimer interface not only reinforces tetramer stability but may also facilitate conformational coupling between subunits, a feature consistent with the observed half‐of‐the‐sites ligand occupancy and the proposed substrate‐gating mechanism. Such inter‐subunit communication is commonly observed in oligomeric hotdog‐fold thioesterases and can influence catalytic efficiency and substrate specificity.

### 2.4 Structures of active site variants

While the elution profiles of the wild‐type and variants indicated that the recombinant proteins were unlikely to be misfolded, we confirmed that the loss of activity was due to loss of active site residues rather than perturbations in the tertiary structure by crystallizing the active site mutants. Similar to the wild‐type enzyme, variants crystallized in several conditions, and where possible, crystals were harvested in different crystallization conditions to confirm that the mutations do not alter the structure, but also that different salt, pH, and/or crystallization packing do not influence the structure. The structures of variants of *Sa*PaaI, Gln32Ala, and Glu47Ala were solved in the P1211 space group; coordinates and structural factors were deposited to PDB and obtained PDB codes 4YBV and 5EP5 respectively. The analysis of tertiary and quaternary structures of variants Gln32Ala and Glu47Ala revealed the similar hotdog architecture. Detailed statistical values of refinement and modeling are listed in Table 1. The analysis of the variant's structures revealed similar hotdog fold conformations to that observed in wild‐type *Sa*PaaI structure, with the greatest RMSD of 0.61 Å (*Sa*PaaI wild type with Gln32Ala) and 0.46 Å (*Sa*PaaI wild type with Glu47Ala) (Figure 1d).

### 2.5 Substrate specificity and active site determination

**FIGURE 2:**
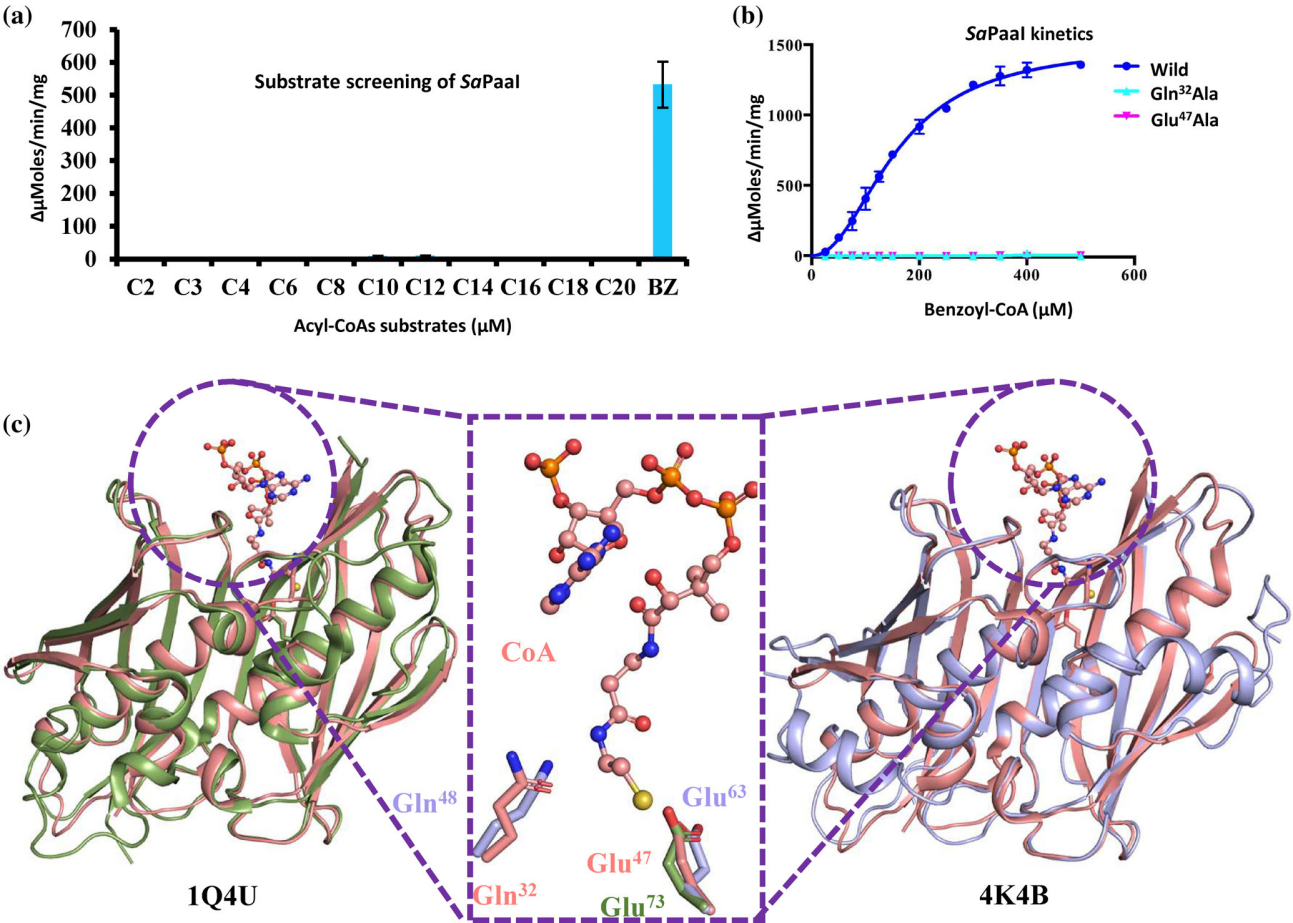
Substrate specificity, kinetic characterization, and active‐site architecture of SaPaaI. (a) Screening of SaPaaI activity against a panel of acyl‐CoA substrates (C2–C20 and benzoyl‐CoA) using a DTNB assay. SaPaaI displays maximal activity toward benzoyl‐CoA. (b) Michaelis–Menten curves for wild‐type SaPaaI and active‐site variants using benzoyl‐CoA as substrate. Mutants Gln32Ala and Glu47Ala show loss of activity. (c) Superposition of SaPaaI (Salmon) with a CoA‐bound homologous thioesterase illustrating the positions of Gln32 and Glu47 relative to the CoA thioester bond. The terminal sulfur atom of CoA (Yellow) is positioned adjacent to the catalytic residues. Structural alignment highlights similarity with AsACT (1Q4U, PaleGreen) and EcACT (4K4B, Slate) active‐site geometry.

To determine the substrate specificity of *Sa*PaaI, a range of fatty acyl‐CoA substrates were screened employing an established enzyme assay with 5,5‐dithiobis‐(2‐nitrobenzoate) (DTNB) (Hunt et al., 2002; Khandokar et al., 2017; Tilton et al., 2004; Wei et al., 2009; Yamada et al., 1996). The screening used 333 μM of substrates ranging in carbon chain length from two (C2) to 20 (C20), with the greatest activity of *Sa*PaaI observed against substrate benzoyl‐CoA (C7‐CoA) (Figure 2a). The *K*_m_ and *K*_cat_ were calculated for *Sa*Paal‐WT and mutants on benzoyl‐CoA (*Sa*Paal‐WT: *K*_m_ = 156 μM and *K*_cat_ = 83 s^−1^) (Figure 2b; Table 3). Importantly, kinetic analysis of *Sa*PaaI‐WT revealed a Hill coefficient (*h*) of 2.12 ± 0.10 (Table 3), indicating positive cooperativity in substrate binding. This result demonstrates functional inter‐subunit communication within the tetramer and supports the structural observation of asymmetric CoA occupancy. Although half‐of‐the‐sites reactivity is often associated with negative cooperativity, it can also arise from conformational coupling or alternating activation states within oligomeric assemblies. The observed positive cooperativity therefore suggests that substrate binding in one active site enhances catalytic competence in neighboring subunits while maintaining structural asymmetry. Similar cooperative behaviors have been reported for other oligomeric thioesterases and regulatory enzymes exhibiting conformational coupling (Koshland et al., 1966; Monod et al., 1965; Pabis et al., 2018).

**TABLE 3:** Kinetic parameters of *Sa*PaaI and mutants.

	Enzyme	*K*_m_ (μM)	*K*_cat_ (S^−1^)	*K*_cat_/*K*_m_ (M^−1^ S^−1^)	*h* (Hill coefficient)
*Sa*PaaI	Wild type	159 ± 6	83.69 ± 2.4	5.26X10^5^	2.1 ± 0.1
	Gln^32^Ala	–	–		–
	Glu^47^Ala	–	–		–

Despite a relatively high degree of structural homology within hotdog containing thioesterases, a diverse range of active site configurations have been reported (Table 4). To better understand both the active site configuration within the *S. aureus* thioesterase and to establish a more general understanding of active sites within thioesterases, we set out to characterize the active site through mutagenesis of putative active site residues. Examination of the active site region, assisted by the x‐ray structure and superposition of homologous thioesterases, revealed the following putative active site residues: Gln32, present in the loop between β2 strand and central α1 helix, and Glu47, positioned in the middle of the central α1 helix of *Sa*PaaI (Figure 2c). To test the contribution of these putative active site residues in catalysis, the following mutants were generated: Gln32Ala and Glu47Ala of *Sa*PaaI. These mutants were recombinantly expressed and purified by affinity and SEC, each of which maintained the same elution on the size exclusion column (Figure S2), indicating the absence of any major aberrations in structure due to the mutations. Based on the homologous mapping of other active sites, it is therefore likely that *Sa*PaaI has differential active site residues (see Table 4), which is similar to thioesterases in the TE11 subfamily.

**TABLE 4:** Variation of active site residues among closely related structures.

	*Z* score	RMSD	PDB	TE subfamily	Active site residues
*Sa*PaaI	27.6	0**	4M20‐A	11	Gln^32^, Glu^47^
	20.6	1.2	1Q4U‐A	11	Gly^65^, Glu^73^
	18.7	1.2	2FS2‐B	13	Gly^53^, Asp^61^
	17.3	1.5	3F5O‐G	8	Asn^50^, Gly^57^, Asp^65^, Ser^83^

Note: ** Indicate no RMSD difference, as it is Chain A of the same structure.

The tertiary structure of *Sa*PaaI is very close to the thioesterase structure from *Arthrobacter* sp. (*As*ACT, PDB ID 1Q4S) and *Escherichia coli* (*Ec*ACT, PDBID 4K4B) (Figure 2c). The structural characterization of *As*ACT showed Gly65 and Glu73 residues and *Ec*ACT showed Gln48 and Glu63 are playing a critical role in carrying out the enzyme activity. The results of our mutagenesis study correlate with the findings of the *As*ACT and *Ec*ACP study, and additionally we showed that Gln32 is also important for enzyme activity (Figure 2). Analysis of the mutants *Sa*PaaI‐Gln32Ala and *Sa*PaaI‐Glu47Ala by SEC was undertaken to confirm the stability of the quaternary form and that mutations did not introduce any structural aberrations.

### 2.6 Comparison with other hotdog‐fold proteins

To place *Sa*PaaI in the broader context of hotdog‐fold thioesterases, structural comparisons were performed using the DALI server (Holm, 2020a; Holm, 2020b; Holm & Laakso, 2016). Several close structural homologues were identified, including *Pa*ACT (PDB ID: 4QD9), *Mt*ACT (PDB ID: 3S4K), and *Ec*ACT (PDB ID: 4K4B). Each exhibits a class‐II hotdog topology (β–β–α–β–β–β–β) with RMSD values below 1.5 Å relative to *Sa*PaaI. The observed Hill coefficient greater than unity (*h* ≈ 2.1; Table 3) provides independent kinetic evidence for functional cooperativity within the *Sa*PaaI tetramer. Such cooperative kinetics are well documented in symmetric oligomeric enzymes where ligand binding to one subunit influences the conformational or catalytic competence of neighboring subunits, often resulting in asymmetric ligand occupancy as observed here (Monod et al., 1965; Koshland et al., 1966; Reed et al., 1982).

**FIGURE 3:**
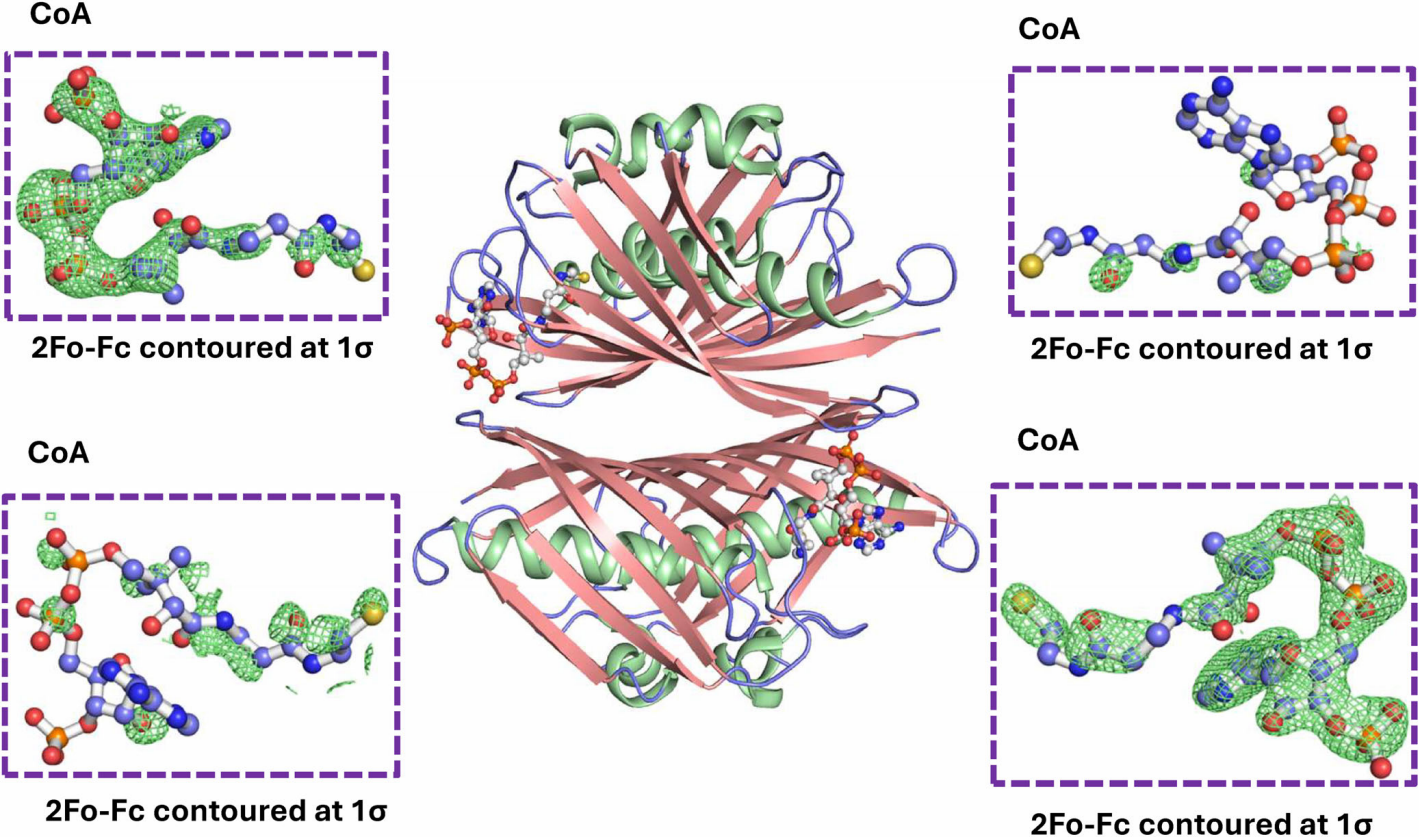
Asymmetric CoA occupancy in the SaPaaI tetramer. The central panel represents the tetrameric assembly of SaPaaI with two CoA molecules bound at opposing monomers. Surrounding panels show 2Fo–Fc electron‐density maps contoured at 1σ around CoA, confirming well‐defined ligand density in two monomers and the absence of density at the equivalent sites in the remaining two monomers. This demonstrates partial ligand occupancy and indicates that SaPaaI crystallizes in an asymmetric ligand‐bound state.

Analysis of the electron density of *Sa*PaaI revealed that only two of the four monomers in the asymmetric unit contained well‐defined density for coenzyme A (CoA). Inspection of 2Fo–Fc maps contoured at 1σ clearly showed CoA in two diagonal monomers, while no interpretable density was observed at the corresponding sites in the remaining two monomers (Figure 3). This demonstrates partial ligand occupancy within the tetramer, indicating that *Sa*PaaI crystallized with two CoA‐bound and two ligand‐free monomers. Such asymmetric occupancy suggests an intrinsic structural bias within the tetramer, likely reflecting distinct conformational states required for ligand engagement and lattice formation.

**FIGURE 4:**
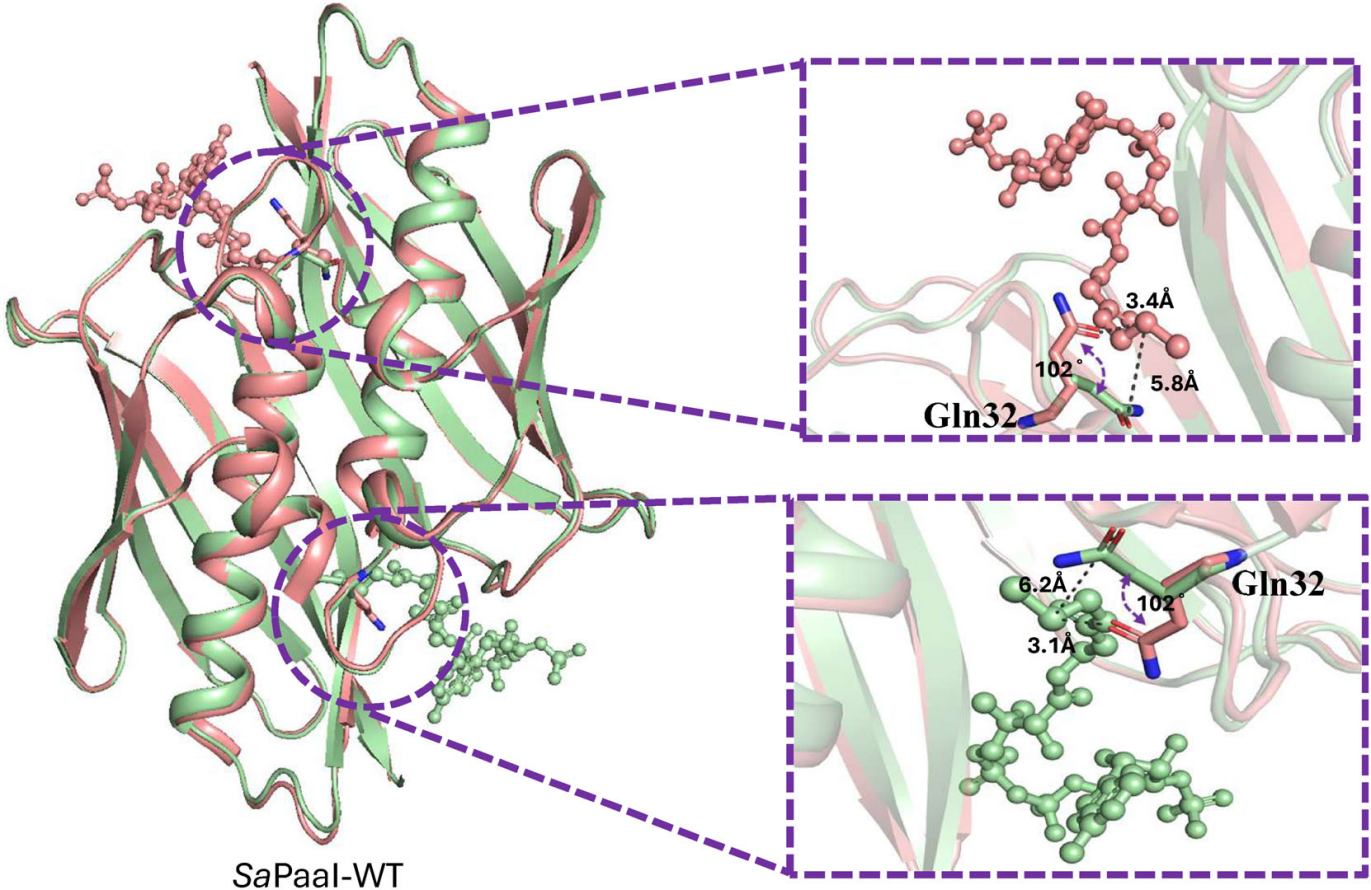
Gln32 conformational changes underpin asymmetric ligand binding in SaPaaI dimers Overlay of the two SaPaaI dimers, AB (Salmon) and CD (PaleGreen), illustrating the asymmetric CoA binding and associated Gln32 conformational changes. In the CoA‐bound monomers, Gln32 rotates ~102° away from the tunnel entrance, creating an open binding site, while in the CoA‐free monomers, Gln32 adopts a closed conformation that occludes the substrate channel. This mutually exclusive arrangement within each dimer supports a half‐of‐the‐sites mechanism in which only one monomer per dimer adopts a ligand‐competent state at a time.

Despite global similarity, variations in the substrate‐binding pocket contribute to diverse substrate specificities across the family. For example, *Pa*ACT, co‐crystallized with benzoyl‐oxydephospho‐CoA (Bod‐CoA), resembles *Sa*PaaI in its preference for aromatic or short‐chain substrates. By contrast, *Ec*ACT features an extended open tunnel that accommodates long‐chain acyl‐CoAs such as C10‐OH‐CoA. The observation that *Sa*PaaI crystallized only in the presence of CoA further supports the idea that ligand binding stabilizes conformational elements surrounding the active site, particularly the β2–α1 loop containing Gln32. Ligand binding likely reduces conformational flexibility of this loop, decreasing structural heterogeneity and thereby promoting stable crystal lattice formation. This ligand‐induced ordering is consistent with the proposed gating mechanism, in which Gln32 undergoes a conformational shift to regulate entry into the substrate‐binding tunnel. Structural overlays reveal that *Sa*PaaI possesses a comparable tunnel, but access is restricted by Gln32, which undergoes a conformational rotation of ~102° upon CoA binding (Figure 4). This movement narrows the tunnel entrance and functions as a molecular gatekeeper, selectively excluding long‐chain substrates.

Importantly, the structural asymmetry observed between the AB and CD dimers further clarifies the mechanistic role of Gln32. In each dimer, only one monomer adopts the ligand‐bound conformation where Gln32 is rotated away from the substrate channel, permitting CoA binding. The adjacent monomer positions Gln32 toward the tunnel entrance, sterically occluding the pocket and preventing substrate access. This mutually exclusive arrangement results in each dimer containing one “active” and one “inactive” site, thereby enforcing a half‐of‐the‐sites configuration that appears intrinsic to the *Sa*PaaI assembly.

The catalytic dyad Gln32‐Glu47 in *Sa*PaaI also differs from the active‐site pairs found in related enzymes, such as Gly65‐Glu73 (*As*ACT) or Gln48‐Glu63 (*Ec*ACT), illustrating evolutionary divergence within TE11‐family thioesterases. These variations demonstrate how fine structural adjustments in the active‐site region and substrate channel geometry allow for specialized substrate recognition across hotdog‐fold enzymes. Together, these comparisons highlight the conserved architectural framework of the hotdog fold while revealing distinct mechanistic adaptations in *Sa*PaaI, particularly the conformational gating role of Gln32, which appears to be unique among characterized homologues.

Notably, comparison with homologous hotdog‐fold thioesterases indicates that while a catalytic Glu residue is broadly conserved across the family, the partnering residue varies (Gly, Gln, or Ser), reflecting evolutionary plasticity in active‐site configuration. The Gln32‐Glu47 dyad in *Sa*PaaI therefore represents a divergent yet functionally competent arrangement that appears adapted for aromatic substrate gating rather than classical catalytic positioning alone.

The asymmetric CoA occupancy observed in the *Sa*PaaI tetramer further suggests a potential regulatory mechanism. Partial ligand occupancy in symmetric oligomers, commonly termed half‐of‐the‐sites reactivity, is a recognized phenomenon in allosteric enzymes, in which only a subset of subunits adopt a ligand‐bound, catalytically competent conformation at any given time (Koshland et al., 1966; Monod et al., 1965). Structural studies of several hotdog‐fold thioesterases also demonstrate ligand‐induced conformational asymmetry. For example, *Thermus thermophilus* PaaI exhibits an asymmetric induced‐fit mechanism in which only two of four catalytic pockets bind substrate in the liganded state (Kunishima et al., 2005), while the *Arthrobacter thioesterase As*ACT undergoes pronounced loop rearrangements upon CoA binding (Song et al., 2012; Thoden et al., 2003).

The structural features of *Sa*PaaI closely mirror these observations. The mutually exclusive conformations of Gln32 within each dimer link substrate occupancy in one monomer to steric modulation of the partner site, providing a structural basis for the half‐of‐the‐sites behavior inferred from CoA density. CoA binding stabilizes the β2–α1 loop containing Gln32, triggering the ~102° rotation proposed to gate substrate entry. The absence of ligand density in the remaining two monomers suggests that these subunits adopt an alternate conformational state within the tetramer. Similar asymmetric ligand binding has been reported in symmetric oligomeric enzymes exhibiting conformational coupling and inter‐subunit communication (Pabis et al., 2018). Which could be addressed via MD simulations in future studies.

Importantly, kinetic analysis revealed a Hill coefficient greater than one, indicating positive cooperativity in substrate binding. This finding supports functional communication between subunits and suggests that substrate binding enhances catalytic competence within the tetrameric assembly while maintaining structural asymmetry. Together, these results indicate that *Sa*PaaI utilizes a ligand‐induced regulatory mechanism involving conformational gating rather than classical negative cooperativity. This behavior aligns with induced‐fit mechanisms described for related hotdog‐fold thioesterases, including the *Streptococcus pneumoniae* PaaI reported by Khandokar et al. (2016), which also exhibits CoA‐induced structural rearrangements and aromatic‐CoA specificity.

*Sa*PaaI represents a previously uncharacterized acyl‐CoA thioesterase from Staphylococcus aureus, and our structural and biochemical findings significantly expand the understanding of hotdog‐fold enzymes within the TE11 subfamily. The high‐resolution crystal structure of *Sa*PaaI reveals a canonical class‐II hotdog fold that assembles as a tetrameric dimer‐of‐dimers, consistent with the oligomerization state observed by SEC. Substrate‐screening experiments demonstrated a pronounced preference for benzoyl‐CoA, indicating selectivity for aromatic substrates rather than long‐chain fatty acyl‐CoAs. Mutagenesis of residues Gln32 and Glu47 resulted in a complete loss of catalytic activity, and crystal structures of the corresponding variants showed no alterations in structure, confirming their essential catalytic roles.

Structural overlays revealed a unique gating mechanism whereby Gln32 undergoes a conformational shift that narrows the substrate tunnel, restricting entry of long‐chain substrates. This regulatory feature has not been previously described in other hotdog‐fold thioesterases. Collectively, these findings establish *Sa*PaaI as a benzoyl‐CoA‐selective thioesterase with a noncanonical catalytic arrangement and a unique gating function.

Aromatic acyl‐CoA species such as benzoyl‐CoA are generated during the catabolism of aromatic amino acids and xenobiotic compounds, and their intracellular accumulation can be toxic to bacterial cells. *Staphylococcus aureus* is known to encounter and metabolize host‐derived aromatic compounds, including phenylalanine and tyrosine derivatives, particularly during infection and tissue colonization. PaaI‐like thioesterases have been implicated in the detoxification and metabolic flux control of such intermediates in multiple bacterial species. In this context, *Sa*PaaI may contribute to the maintenance of aromatic‐CoA homeostasis during host colonization or under stress conditions encountered during infection.

Given the role of aromatic‐CoA intermediates in bacterial metabolism and the importance of thioesterase‐mediated detoxification pathways, *Sa*PaaI may represent a potential antimicrobial target. Inhibitors designed to stabilize the closed Gln32 conformation could, in principle, restrict aromatic‐CoA turnover in *S. aureus*, thereby disrupting metabolic homeostasis under stress or host‐associated conditions. These structural insights may provide a framework for future inhibitor development targeting aromatic‐acyl‐CoA metabolism in pathogenic bacteria.

## 3 CONCLUSIONS

In this study, we present the first structural and functional characterization of the *Staphylococcus aureus* thioesterase *Sa*PaaI. The enzyme adopts a tetrameric class‐II hotdog fold with a well‐defined substrate‐binding tunnel and exhibits a strong preference for benzoyl‐CoA over longer‐chain fatty acyl‐CoAs. Through structural analysis, mutagenesis, and kinetic measurements, we identified Gln32 and Glu47 as essential catalytic residues. Comparative structural analysis revealed distinct active‐site geometry and a conformationally responsive gatekeeper residue (Gln32) that restricts substrate access. The cooperative kinetic behavior observed for *Sa*PaaI, reflected by a Hill coefficient greater than two, is consistent with the asymmetric ligand occupancy and half‐of‐the‐sites architecture revealed by crystallographic analysis. These findings position *Sa*PaaI as an important representative of TE11‐family thioesterases.

## 4 METHODS

### 4.1 Gene cloning and protein expression

The gene encoding *Sa*PaaI (SAV0944) from *Staphylococcus aureus* subsp. *aureus* Mu50 was amplified by PCR and cloned into the pET‐28a(+) vector using N‐terminal His₆‐tag and TEV protease cleavage site. The plasmid is transformed into *Escherichia coli* BL21(DE3) competent cells for recombinant expression. A single colony was used to inoculate LB medium containing 50 μg/mL kanamycin and grown at 37°C with shaking to an OD₆₀₀ of 0.6–0.8. Protein expression was induced with 0.5 mM IPTG, and cultures were incubated overnight at 18°C.

### 4.2 Protein purification

Cells were harvested by centrifugation (5000×*g*, 20 min, 4°C) and resuspended in lysis buffer (20 mM Tris–HCl, pH 8.0; 300 mM NaCl; 10 mM imidazole; 5 mM β‐mercaptoethanol). Cells were lysed by sonication, and the lysate was clarified by centrifugation (20,000×*g*, 30 min, 4°C). The supernatant was loaded onto Ni^2+^‐NTA affinity resin pre‐equilibrated with lysis buffer. Bound protein was washed with buffer containing 30 mM imidazole and eluted with 250 mM imidazole. The 6x His tag was removed by overnight incubation with TEV protease at a 1:50 (w/w) protease‐to‐protein ratio during dialysis against 20 mM Tris–HCl pH 8.0, 150 mM NaCl, and 2 mM DTT. The cleaved protein was passed again over Ni^2+^‐NTA resin to remove uncleaved protein, the His₆‐tag fragment, and TEV protease. Final purification was achieved using size‐exclusion chromatography (SEC) on a Superdex 200 16/60 column (GE Healthcare) equilibrated in SEC buffer (20 mM Tris–HCl pH 8.0, 150 mM NaCl, 2 mM DTT). Fractions corresponding to tetrameric *Sa*PaaI were pooled and concentrated using a 10 kDa MWCO centrifugal concentrator. Protein concentration was measured using the absorbance at 280 nm and predicted extinction coefficient. Approximately 14 mg of soluble, highly purified protein was obtained from 1 L of bacterial culture. Protein integrity and purity were confirmed by SDS–PAGE before crystallization trials.

### 4.3 Site‐directed mutagenesis

Mutations Gln32Ala and Glu47Ala were generated by PCR‐based site‐directed mutagenesis using complementary primers carrying the desired nucleotide substitutions. The mutant constructs were verified by Sanger sequencing. Expression and purification of the mutant proteins were performed as described for wild‐type *Sa*PaaI.

### 4.4 Crystallization

Initial attempts to crystallize *Sa*PaaI in the absence of ligand were unsuccessful. Crystals formed only when coenzyme A (CoA) was included in the protein solution prior to setting up crystallization drops. Final crystallization conditions therefore used *Sa*PaaI pre‐incubated with CoA, indicating that ligand binding stabilizes the protein sufficiently for lattice formation (Khandokar et al., 2014). Purified *Sa*PaaI was concentrated to 10–12 mg/mL for crystallization trials. Initial screening was performed using commercial sparse‐matrix screens (JCSG+, PACT, and Crystal Screen) in 96‐well sitting‐drop vapor‐diffusion format at 18°C. Optimized crystals were obtained in conditions containing PEG 4000, Sodium Citrate, and Propanol in presence of Coenzyme A. Drops consisted of 1 μL protein mixed with 1 μL reservoir solution and equilibrated against 500 μL reservoir. Crystals typically appeared within 2–5 days and reached full size within 1 week.

Final crystallization screening was performed using the hanging‐drop vapor‐diffusion method with commercial sparse‐matrix screens. Only a limited number of conditions produced crystals, and optimization yielded the final crystallization condition used for structure determination: 0.1 M sodium citrate tribasic dihydrate pH 5.6, 20% (v/v) 2‐propanol, and 20% (w/v) polyethylene glycol (PEG) 4000. Crystals suitable for diffraction appeared under this condition and were used to collect complete x‐ray diffraction datasets at the MX2 beamline, Australian Synchrotron (Clayton, Australia).

### 4.5 X‐ray data collection and processing

Crystals were cryoprotected with reservoir solution supplemented with 20–25% (v/v) glycerol and flash‐cooled in liquid nitrogen. Diffraction data were collected at the MX2 beamline of the Australian Synchrotron (Clayton, Australia). Data were processed using XDS, and scaling and merging were performed using Aimless from the CCP4 suite. Data quality statistics, including resolution, completeness, redundancy, Rmerge, and CC1/2, were evaluated to assess dataset suitability.

### 4.6 Structure solution and refinement

The *Sa*PaaI structure was solved by molecular replacement using Phaser with an appropriate homologous hotdog‐fold thioesterase model as the search template. Iterative model building was carried out in Coot, and refinement was performed using Phenix.refine with TLS and restrained refinement. Water molecules were added automatically and verified manually. The geometry of the final model was validated using MolProbity, with Ramachandran statistics and clash scores used to ensure model quality.

### 4.7 Size‐exclusion chromatography for oligomeric state analysis

To determine the oligomeric state, wild‐type and mutant *Sa*PaaI proteins were analyzed using analytical SEC on a Superdex 200 Increase 10/300 GL column equilibrated in 20 mM Tris–HCl pH 8.0, 150 mM NaCl, and 2 mM DTT. Molecular‐weight standards were used to generate a calibration curve. Elution volumes corresponding to wild‐type and mutant proteins were compared to infer oligomeric assembly.

### 4.8 Enzyme activity assays

Thioesterase activity was measured using a panel of acyl‐CoA substrates, including benzoyl‐CoA, following standard spectrophotometric methods. Assays were conducted in 50 mM Tris–HCl pH 7.5 at 25°C. Reactions were initiated by adding *Sa*PaaI (wild‐type or mutant) to substrate‐containing buffer, hydrolysis was monitored by the decrease in absorbance at 260 nm corresponding to the release of CoA. Kinetic parameters were determined by fitting initial rates to the Michaelis–Menten equation using GraphPad Prism. Mutant variants were assayed under identical conditions to determine the impact of residue substitution on catalytic activity.

### 4.9 Structural analysis and comparative modeling

Structural superpositions, analysis of the substrate‐binding tunnel, and visualization of catalytic residues were performed using PyMOL and Coot. Sequence alignments with homologous thioesterases were generated using Clustal Omega. Tunnel geometries and cavity volumes were analyzed using CAVER and PyMOL cavity tools.

## AUTHOR CONTRIBUTIONS

Yogesh Khandokar: Investigation, original draft, conceptualization, methodology, writing—review and editing. Parul Srivastava, Renate H. M. Schwab, Ashish Sethi, and Naveen Vankadari: Investigation, methodology, writing—review and editing. Jade K. Forwood: Supervision, project administration, writing—review and editing.

## CONFLICT OF INTEREST STATEMENT

The authors declare no conflicts of interest.

## Supporting information


**Figure S1.** Affinity and analytical size‐exclusion chromatography of S*a*PaaI. (A) AKTA profile showing the His tagged SaPaaI elution profile using prepack Ni NTA column with an imidazole gradient (B) SEC (10/300 S75) Elution profile of wild‐type SaPaaI (blue) corresponding to molecular weight of tetrameric form of protein.
**Figure S2.** Analytical size‐exclusion chromatography of SaPaaI. Elution profiles of wild‐type S*a*PaaI (blue), Gln32Ala (red), and Glu47Ala (green). All samples elute as single, symmetric peaks at volumes consistent with tetrameric assembly, indicating that mutations do not perturb quaternary structure.
**Figure S3.** (A) SaPaaI crystal used for data collection on MX2 beamline and (B) showing its diffracting to 2 Å

## Data Availability

The authors confirm that all data underlying the findings are fully available without restriction. All relevant data are within the paper and its Supporting Information files.
