# Supplementary material for: Tetrameric hotdog‐fold structure and catalytic mechanism of the Sa PaaI thioesterase from Staphylococcus aureus
Source: Protein Sci. 2026 Apr 27;35(5):e70583. doi: 10.1002/pro.70583 (PMC13114782; doi:10.1002/pro.70583)
Supplement: Supplementary file 1 — Figure S1. Affinity and analytical size‐exclusion chromatography of SaPaaI. (A) AKTA profile showing the His tagged SaPaaI elution profile using prepack Ni NTA column with an imidazole gradient (B) SEC (10/300 S75) Elution profile of wild‐type SaPaaI (blue) corresponding to molecular weight of tetrameric form of protein. Figure S2. Analytical size‐exclusion chromatography of SaPaaI. Elution profiles of wild‐type SaPaaI (blue), Gln32Ala (red), and Glu47Ala (green). All samples elute as single, symmetric peaks at volumes consistent with tetrameric assembly, indicating that mutations do not perturb quaternary structure. Figure S3. (A) SaPaaI crystal used for data collection on MX2 beamline and (B) showing its diffracting to 2 Å [file PRO-35-e70583-s001.pdf]

Supporting Figure 1

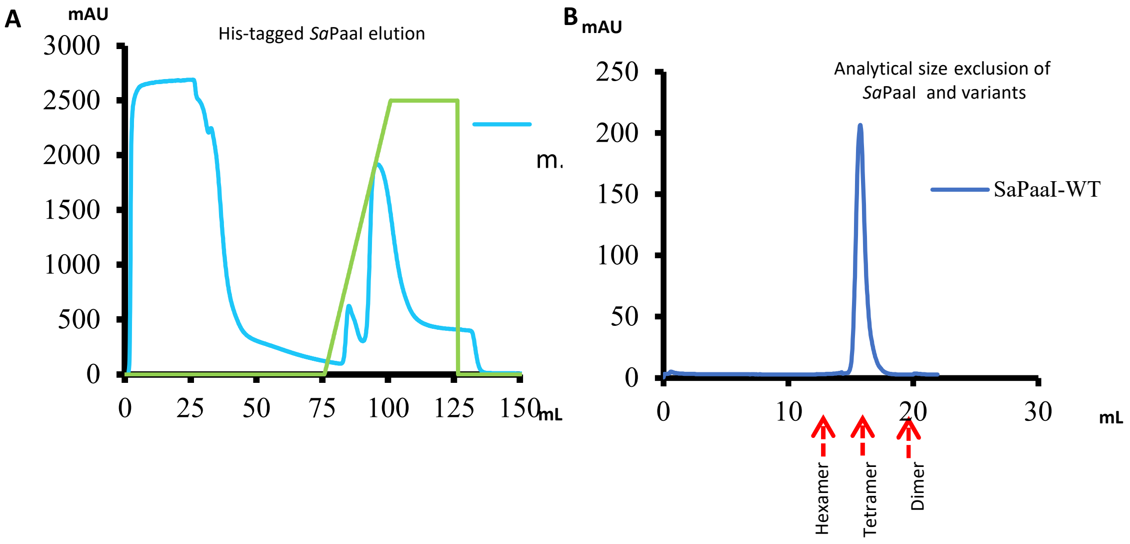

Affinity and analytical size-exclusion chromatography of *Sa*PaaI.

(A) AKTA profile showing the His tagged SaPaaI elution profile using prepack Ni-NTA column with an imidazole gradient

(B) SEC (10/300 S75) Elution profile of wild-type *Sa*PaaI (blue) corresponding to molecular weight of tetrameric form of protein

Supporting Figure 2

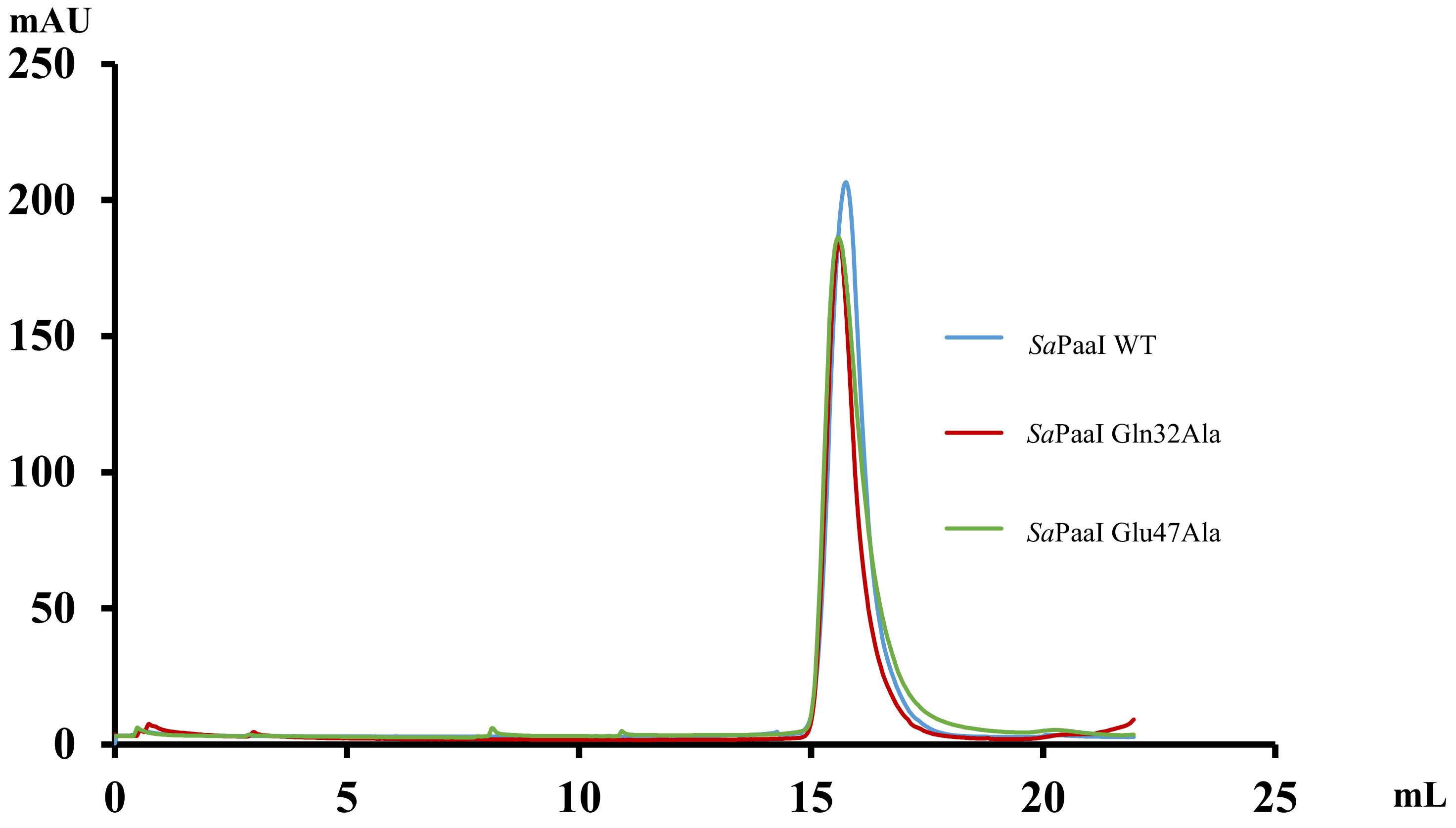

Analytical size-exclusion chromatography of SaPaaI.
Elution profiles of wild-type *Sa*PaaI (blue), Gln32Ala (red), and Glu47Ala (green). All samples elute as single, symmetric peaks at volumes consistent with tetrameric assembly, indicating that mutations do not perturb quaternary structure.

Supporting Figure 3

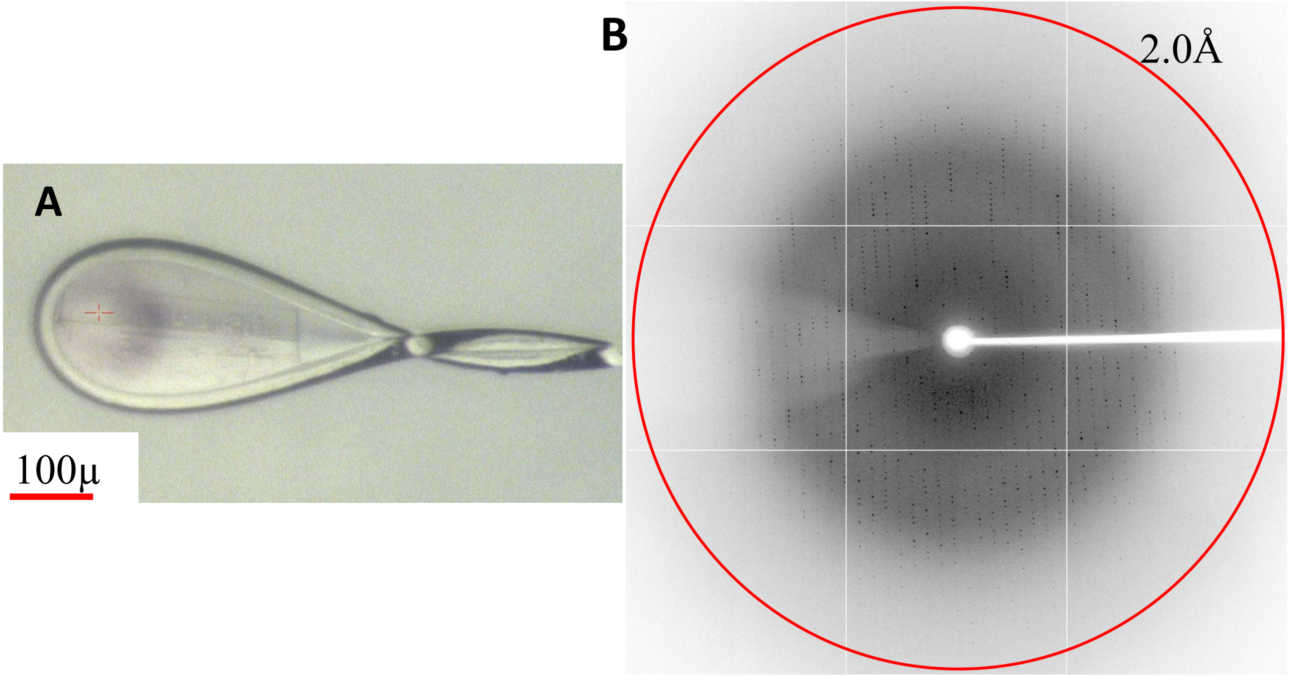

A. SaPaaI crystal used for data collection on MX2 beamline and **B.** showing its diffracting to 2Å
